# Antiretrovirals Promote Insulin Resistance in HepG2 Liver Cells through miRNA Regulation and Transcriptional Activation of the NLRP3 Inflammasome

**DOI:** 10.3390/ijms24076267

**Published:** 2023-03-27

**Authors:** Jivanka Mohan, Terisha Ghazi, Makabongwe S. Mazibuko, Anil A. Chuturgoon

**Affiliations:** Discipline of Medical Biochemistry, School of Laboratory Medicine and Medical Sciences, College of Health Sciences, University of KwaZulu-Natal, Durban 4041, South Africa

**Keywords:** antiretrovirals, metabolic syndrome, insulin resistance, inflammasome, miR-128a

## Abstract

Metabolic syndrome (MetS) is a non-communicable disease characterized by a cluster of metabolic irregularities. Alarmingly, the prevalence of MetS in people living with Human Immunodeficiency Virus (HIV) and antiretroviral (ARV) usage is increasing rapidly. Insulin resistance is a common characteristic of MetS that leads to the development of Type 2 diabetes mellitus (T2DM). The progression of insulin resistance is strongly linked to inflammasome activation. This study aimed to draw links between the combinational use of Tenofovir disoproxil fumarate (TDF), Lamivudine (3TC), and Dolutegravir (DTG), and inflammasome activation and subsequent promotion of insulin resistance following a 120 h treatment period in HepG2 liver in vitro cell model. Furthermore, we assess microRNA (miR-128a) expression as a negative regulator of the IRS1/AKT signaling pathway. The relative expression of phosphorylated IRS1 was determined by Western blot. Transcript levels of NLRP3, IL-1β, JNK, IRS1, AKT, PI3K, and miR-128a were assessed using quantitative PCR (qPCR). Caspase-1 activity was measured using luminometry. Following exposure to ARVs for 120 h, NLRP3 mRNA expression (*p* = 0.0500) and caspase-1 activity (*p* < 0.0001) significantly increased. This was followed by a significant elevation in IL-1β in mRNA expression (*p* = 0.0015). Additionally, JNK expression (*p* = 0.0093) was upregulated with coinciding increases in p-IRS1 protein expression (*p* < 0.0001) and decreased IRS1 mRNA expression (*p* = 0.0004). Consequently, decreased AKT (*p* = 0.0005) and PI3K expressions (*p* = 0.0007) were observed. Interestingly miR-128a expression was significantly upregulated. The results indicate that combinational use of ARVs upregulates inflammasome activation and promotes insulin resistance through dysregulation of the IRS1/PI3K/AKT insulin signaling pathway.

## 1. Introduction

MetS is classified by the World Health Organization (WHO) as a global public hazard that affects 20–30% of adults. The non-communicable disease can be classified by several different pathologies, including insulin resistance, obesity, arterial hypertension, and dyslipidaemia [1,2]. These isolated pathologies may contribute to the development of more severe conditions such as Type 2 diabetes mellitus (T2DM) and cardiovascular diseases (CVD) [2].

The literature indicates a strong correlation between the occurrence of MetS in people living with HIV (PLWH) [3,4,5]. HIV severely affects a considerable portion of the global population, with greater specificity in Sub-Saharan Africa. By the end of 2019, roughly 38 million people worldwide were living with HIV, with 1.7 million new infections for the year [6]. Of these statistics, 7.8 million PLWH were localised to South Africa [7].

Of the total infected global population, 26 million had access to antiretroviral (ARV) treatment at the end of 2019 [6]. Highly active antiretroviral therapy (HAART) has significantly decreased the HIV-infected population’s mortality [8,9]. However, clinical studies have indicated that the usage of HAART promotes MetS in PLWH [10], with at least 21% displaying insulin resistance [11,12,13]. Despite these findings, proper mechanisms of action surrounding the combinational usage of HAART remain elusive.

Insulin signaling is imperative for glucose uptake in muscular and skeletal cells [14]; however, in the liver, insulin is responsible for initiating fatty acid synthesis through the regulation of de novo lipogenesis [15]. Aberrations in the insulin signaling pathway lead to fatty acid accumulation and the progression of non-alcoholic fatty liver disease (NAFLD) [16]. The latter is closely linked with the development of T2DM and other metabolic complications [17].

It is well understood that inflammation is strongly linked to the occurrence of insulin resistance [18]. The upregulation of inflammatory genes and proteins leads to the serine phosphorylation of the insulin receptor I (IRS1), which has several downstream targets that reduce insulin sensitivity [19]. More specifically, increased phosphorylated IRS1 (p-IRS1) causes decreased expression of Protein kinase B (AKT), and phosphoinositide 3-kinase (PI3K) allows for the progression of insulin resistance [20].

Aside from the more common pro-inflammatory cytokines, the (NOD-like) pyrin domain containing 3 (NLRP3) inflammasome has gained popularity for its implications in insulin resistance [21]. This is observed through the cleavage of pro- interleukin 1β to interleukin- 1β (IL-1β), which allows for the phosphorylation of IRS1 both directly and indirectly [19]. The occurrence of the NLRP3 inflammasome in PLWH has been well studied; however, mechanisms surrounding combinational ARV usage and possible activation of inflammasomes and its linkage to insulin resistance remain limited [22].

Additionally, insulin resistance can be regulated epigenetically through the expression of miRNAs [23]. Increased expression of specific miRNAs results in decreased expression of targets related to insulin resistance. In this study, we focus on miR-128a, which is known to negatively regulate the IRS1/AKT axis, thus promoting insulin resistance [24].

In 2016, the WHO suggested the usage of TDF, 3TC, and emtricitabine (FTC)/efavirenz (EFV) as the preferred combinational treatment for HIV in young adolescents and adults. However, as research progressed, the WHO updated its recommendations. Guidelines published in 2018 recommended DTG for use in first-line treatment leading to combinational treatment of 3TC, TDF, and DTG being popularised following approval by the WHO [25]. Studies often assess the side effects of these drugs in isolation, with very few studies evaluating biochemical mechanisms involved in their combinational usage [26]. This study aimed to understand the relationship between the combinational use of TDF, 3TC and DTG and inflammasome activation and its promotion of insulin resistance in liver cells following prolonged in vitro exposure. HepG2 cells were used, as they mimic the physiological functions and epigenetic profiles of primary hepatocytes [27]. Additionally, they have commonly been used as in vitro models for studies assessing ARV toxicity [28,29,30]. Following an extensive review of the literature, a treatment period of 120 h (h) was selected to assess the chronic effects of ARVs in HepG2 cells. This duration is often used in ARV in in vitro studies [28,29].

Furthermore, we highlight miRNA regulation and its possible implications for the progression of insulin resistance. Evidence from this study can be used to develop therapies with reduced side effects related to MetS.

## 2. Results

### 2.1. Combinational Usage of ARVs Results in the Upregulation of Key Components of the Inflammasome Pathway

**Figure 1:**
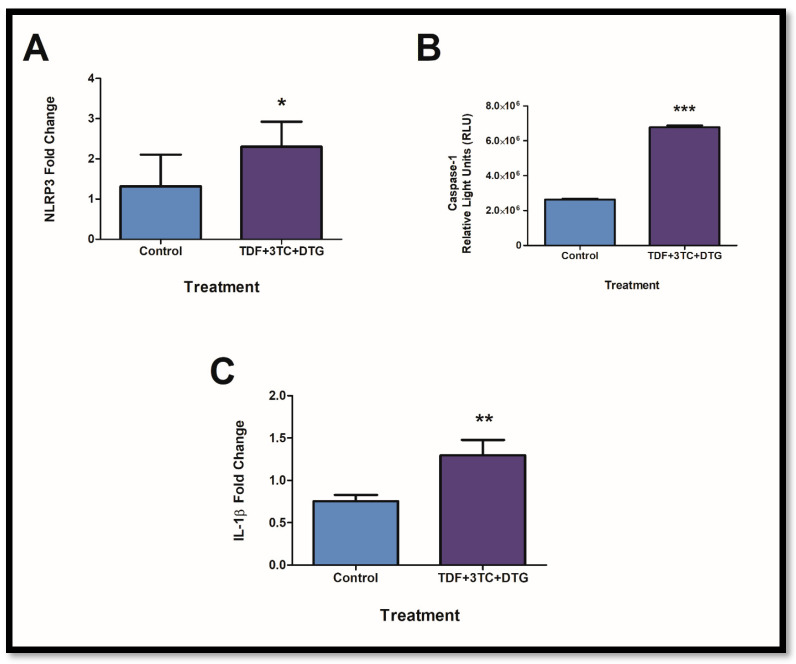
ARVs increase the expression and activity of components in the NLRP3 inflammasome. *NLRP3* mRNA expression was significantly increased ((**A**); * *p* < 0.05). Additionally, caspase-1 activity showed significant upregulation following exposure ((**B**); *** *p* < 0.0001). *IL-1β* mRNA showed significant elevations in expression ((**C**); ** *p* < 0.005).

The main components of the inflammasome pathway were assessed to determine activation following prolonged exposure. NLRP3 results in several downstream actions that cleave procaspase-1 to caspase-1. This ultimately leads to the activation of IL-1β from pro-IL-1β. Following exposure to ARVS, *NLRP3* mRNA expression was significantly increased (Figure 1A; *p* = 0.0500) with resulting increases in caspase-1 activity (Figure 1B; *p* =< 0.0001). This was accompanied by increased expression of *IL-1β mRNA* (Figure 1C; *p* = 0.0015).

### 2.2. Exposure to ARVs Alters JNK and Insulin Receptor Expressions

**Figure 2:**
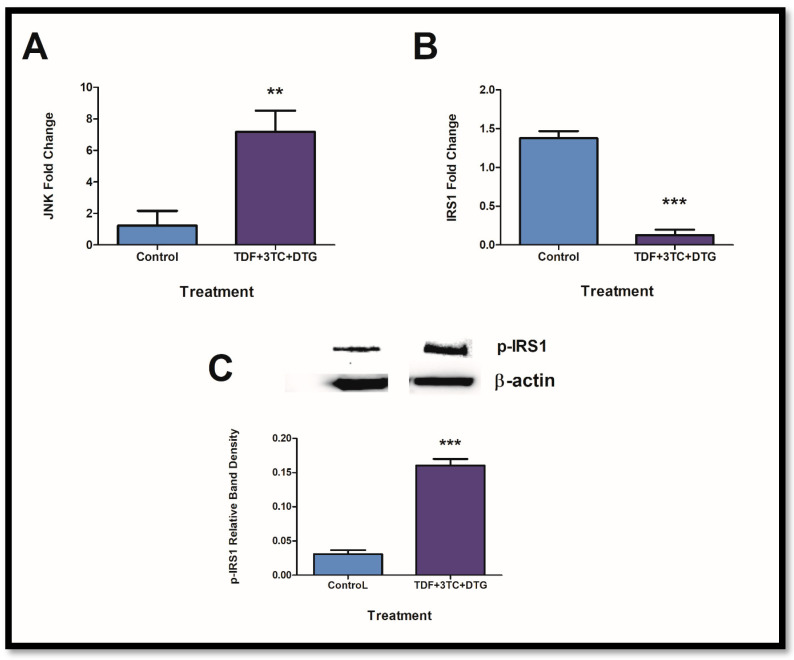
ARVs interfere with insulin receptor expressions via JNK. *JNK* expression was significantly increased ((**A**); ** *p* < 0.005) whereas *IRS1* expressions showed suppression ((**B**); *** *p* < 0.0001). Furthermore, phosphorylated IRS1 protein expression showed significant elevations ((**C**); *** *p* < 0.0001). Bands for protein expression of p-IRS1 and β-actin were obtained from the same membrane.

The expression of *IRS1* and intermediates was assessed after confirmation of inflammasome activity. IL-1β can lead to JNK activation and a decrease in IRS1 expression. JNK allows for the phosphorylation of present IRS1 to yield p-IRS1. The latter is responsible for the progression of insulin resistance. After exposure to ARVs, *JNK* mRNA expression significantly increased (Figure 2A; *p* = 0.0093) with resulting decreases in *IRS1* mRNA expression (Figure 2B; *p* = 0.0004). Furthermore, p-IRS1 showed increased protein expression (Figure 2C: *p* < 0.0001).

### 2.3. Prolonged Exposure to ARVs Resulted in Disruption of the PI3K/AKT Pathway via Inflammasome Activation and miR-128a Expression

**Figure 3:**
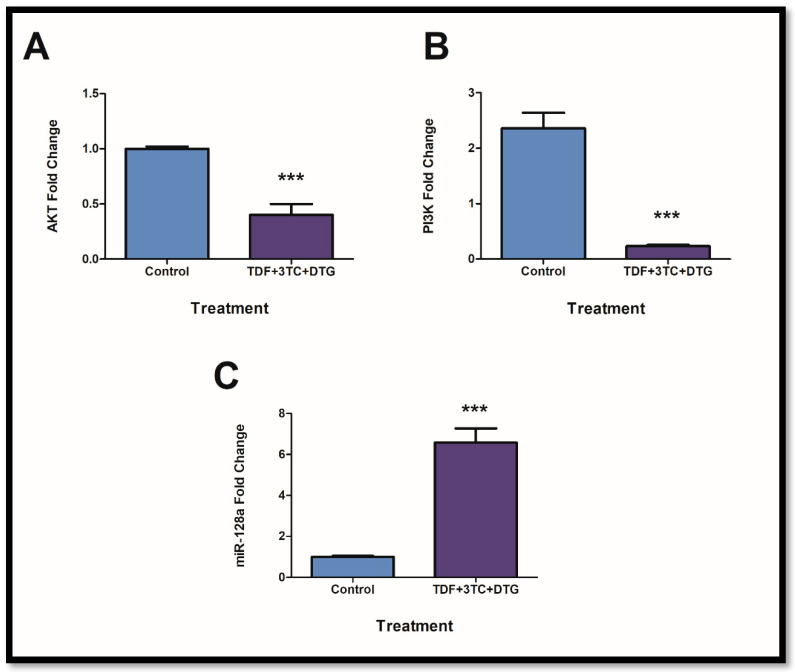
PI3K/AKT axis was disrupted through inflammasome activation and miRNA regulation. *AKT* ((**A**); *** *p* < 0.0001) and *PI3K* ((**B**); *** *p* < 0.0001) mRNA expression showed significant decreases. Conversely, miR-128a was elevated ((**C**); *** *p* < 0.0001).

Elevation in p-IRS1 expressions coincides with decreased AKT and PI3K expression. Such effects result in insulin resistance promotion. Furthermore, miR-128a is known to regulate the expression of AKT and IRS1 negatively. Following exposure, both *AKT* (Figure 3A; *p* = 0.0005) and *PI3K* (Figure 3B; *p* = 0.0007) were significantly reduced, whereas the expression of miR-128a showed significant elevation (Figure 3C; *p* = 0.0002).

## 3. Discussion

MetS is a non-communicable disease that is diagnosed by having one or more of a cluster of metabolic irregularities. Alarmingly, the prevalence of MetS in PLWH and ARV usage is increasing rapidly. Older generations of ARVS were associated with severe side effects that resulted in patients discontinuing use [26,28]. Fortunately, newer generations of drugs have fewer side effects but are still associated with metabolic complications [31]. The WHO has stressed the need for developing new ARVs and phasing in newer generations of ARVs to ensure side effects are manageable and reduce HIV drug resistance [8,25]. This study aimed to look at biochemical mechanisms and epigenetic modifications associated with ARVs and MetS in liver cells which originate from the metabolic hub of the human body. Evidence from this study will aid in understanding possible mechanisms associated with ARV usage and insulin resistance. More specifically, we highlight the role of the NLRP3 inflammasome in the progression of insulin resistance and miRNA regulation of targets associated with insulin resistance/sensitivity.

**Figure 4:**
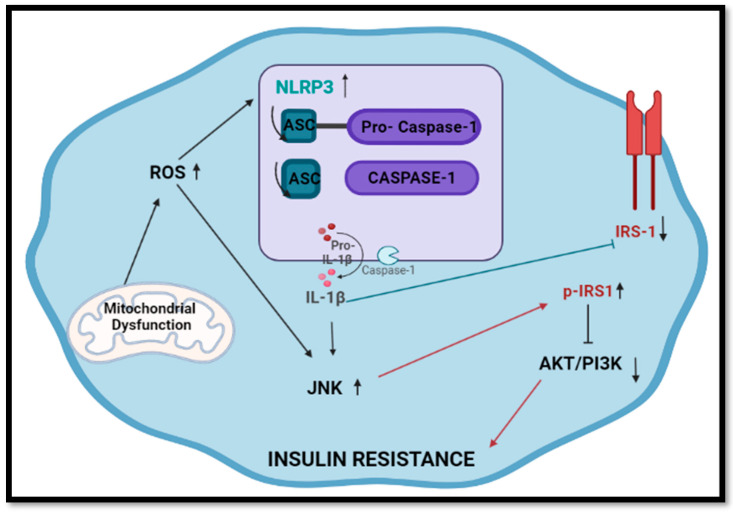
Inflammasome activation and subsequent occurrence of insulin resistance. Following activation of inflammasomes via stress, IL-1β allows for JNK activation leading to increases in p-IRS1 and aberration in AKT/PI3K signaling. Furthermore, decreased expression of IRS1 occurs.

Previous evidence has shown links between inflammasome activation and the constant pro-inflammatory states associated with HIV infection [22,32]. Little evidence exists to show the combinational use of ARVs and inflammasome activation. Inflammasomes are multimeric protein complexes that assemble in response to different stressors. Several different types of inflammasomes exist with similar functions and different response stimuli [33,34]. The NLRP3 inflammasome is mostly activated in response to mitochondrial stress (Figure 4). Upon stimulation, the NLRP3 proteins bind to the ASC proteins via pyridinoline interactions [35,36,37]. Pro-caspase-1 then interacts with the ASC protein via CARD domains, which ultimately leads to the auto-proteolytic maturation of pro-caspase-1 into active caspase-1 [37]. The latter allows for pro-inflammatory cytokines into their bioactive forms. More specifically, pro-IL-1β is cleaved to IL-1β and can perform inflammatory functions [33].

The current study shows significant increases in NLRP3 gene expression and caspase-1 activity. Additionally, IL-1β mRNA expression showed substantial elevations. The evidence presented coincides with the upregulation of inflammasome at a transcriptional level. The ARVs tested have previously been associated with increased ROS production and mitochondrial stress. More specifically, DTG has been implicated in the rise in ROS production via the dysregulation of Ca^2+^ signaling [38]. Aside from this, studies in rat liver and kidneys showed that using TDF and 3TC increased lipid peroxidation (a consequence of ROS production) and depleted glutathione levels when paired with Efavirenz [39]. These are common markers for mitochondrial dysfunction. The ability of the ARVs tested to induce ROS production provides plausible reasoning for the increase of genes and components related to the NLRP3 inflammasome. Despite the individual ARVs being unable to cause a significant increase in NLRP3 gene expression (Supplementary Figure S1A), combinational usage caused considerable elevation. This is possibly attributed to synergistic effects in mitochondrial dysfunction observed in previous studies [38,39].

In in vitro work, IL-1β suppresses insulin sensitivity by increasing JNK-dependent serine phosphorylation of IRS1 (Figure 4). Subsequently, increased p-IRS1 causes aberrations in insulin-induced PI3K/Akt signaling in cells [19]. Aside from activation via IL-1β, JNK can be upregulated by detecting excessive ROS production and mitochondrial dysfunction [20]. The present study shows a significant increase in *JNK* expression following exposure. Furthermore, p-IRS1 protein expression increased, coinciding with the increases in *JNK*. Aside from this, previous studies show correlations between increased IL-β expression and decreased IRS1 expression [19], correlating with data from the present study. Similarly, singular ARV treatment produced no significant change in JNK expression (Supplementary Figure S2A); however, combinational usage prompted responses. Studies have shown that DTG can reduce mitochondrial ATP production and redox activity in murine cells, further providing reasoning for JNK activation [40]. In similar studies using rat kidneys, TDF was found to reduce ATP production via action on electron transport chain complexes signaling for aberrant mitochondrial metabolism [41]. The individual capacity of these drugs to initiate mitochondrial dysfunction [41,42] provides reasoning for their synergistic activation of related targets such as *JNK* and, subsequently, *IRS1* gene expression.

Under typical conditions, IRS1 allows for the activation of PI3K and AKT. The latter intermediates allow for an increase in glucose uptake, vasodilation, and insulin secretion in cells. However, elevated expression of p-IRS1 as a consequence of serine phosphorylation by JNK causes a decrease in PI3K and AKT expression, thus promoting the occurrence of insulin resistance and, ultimately, T2DM if not controlled [20]. The current study showed significant decreases in *AKT* and *PI3K* expression, coinciding with the observed elevation of p-IRS1 expression. Previous literature indicates that DTG can promote insulin resistance in adipose tissue through the induction of oxidative stress; however, mechanisms remained unclear [23]. In earlier studies, using 3TC with other ARVs caused disturbances in glucose metabolism with a significant decrease in insulin-mediated glucose disposal, thus showing the promotion of insulin resistance. However, no biochemical mechanism of action was established [43]. The current study provides possible mechanisms for the occurrence of insulin resistance at a genomic and protein level following combinational usage.

Aside from this, the evidence suggests that the tested ARVS can promote insulin resistance through epigenetic changes. It is well known that miRNAs can negatively regulate the expression of specific targets [44]. We assessed the expression of miR-128a, which was found to negatively regulate IRS1/AKT signaling in previous studies [24]. This occurs when miR-128a binds to the 3′-untranslated region (3′-UTR) of target mRNA [24]. Following exposure in the current study, miR-128a was significantly increased while *AKT* and *IRS1* showed correlating decreases. Individual exposure showed no significant changes (Supplementary Figure S3C). The data suggest that combinational ARVs can promote insulin resistance through the upregulation of miRNA expression. This has implications for future studies that are imperative to understanding epigenetic changes induced by ARV exposure. At present, studies showing combinational use and epigenetic modifications remain limited.

The current study highlights the transcriptional activation of inflammasomes and provides insights into the possible mechanism of insulin resistance. Previous studies use transcript levels as a suitable indicator for promotion of inflammasome activation [45] and insulin resistance [46,47]. This information is useful for future in vivo studies that may evaluate more detailed markers.

Furthermore, we show miRNA regulation that coincided with insulin resistance promotion in cells. Combinational usage showed an increase in inflammasome-related genes and enzymes, resulting in reduced IRS1 signaling and, subsequently, promotion in insulin resistance despite the drugs not achieving the same result during individual exposure.

## 4. Materials and Methods

### 4.1. Materials

Antiretroviral drugs were obtained from the NIH AIDS reagents program. HepG2 cells were purchased from American Type Culture Collection (Johannesburg, South Africa). Cell culture media and supplements were purchased from Lonza (Basel, Switzerland). Luminometry kits were obtained from Promega (Madison, WI, USA). Western blot reagents were purchased from Bio-Rad (Hercules, CA, USA). Unless otherwise stated, all remaining reagents were obtained from Merck (Darmstadt, Germany).

Cell culture and treatment: HepG2 cells were cultured in 25 cm^3^ cell culture flasks using CCM [Eagle’s minimum essentials medium (EMEM) supplemented with 10% foetal calf serum, 1% pen-strep-fungizone, and 1% L-glutamine] and maintained in a humidified incubator (37 °C, 5% CO_2_) until approximately 70% confluent. All treatments were carried out using cells from the same passage to avoid any discrepancies in data (Passage number: 3).

Cells were then exposed to physiological concentrations (C_max_- maximum plasma concentration) of ARVs (3TC: 1.51 µg/mL, TDF: 0.3 µg/mL, DTG: 3.67 µg/mL) [48,49,50] for 120 h as per Nagiah et al., 2015 [28]. ARVs were dissolved in phosphate buffered saline (PBS) for treatment. Cells were washed every 24 h with 0.1 M PBS and fresh CCM with ARVs were added to flasks. Untreated cells containing CCM only were used for controls. Further assays were carried out following treatment as explained above.

### 4.2. Caspase 1 Detection

Caspase-1 activity was measured using the Caspase-Glo^®^ 1 Inflammasome Assay (G9951, Promega, Madison, WI, USA). Following incubation cells with treatment, 50 µL of cell suspension (20,000 cells/well in 0.1 M PBS) was added into an opaque microtitre plate in triplicate. The Caspase-Glo^®^ 1 reagents were reconstituted as per manufacturer’s guidelines and 50 µL was added to each well containing cells. Plates were then incubated (dark, 1 h, Room temperature (RT)). Luminescence was measured using a Modulus™ Microplate Reader (Turner Biosystems, Sunnyvale, CA, USA). Results were expressed as relative light units (RLU).

### 4.3. Western Blot

Following 120 h treatment of HepG2 cells with ARVs, cells were washed with 0.1 M PBS. Thereafter, 150 µL Cytobuster™ Reagent was added to each flask (Novagen, San Diego, CA, USA, catalogue no. 71009) and incubated on ice for 30 min (min). Mechanical lysis of cells was performed using a cell scraper, and contents were transferred to 1.5 mL micro-centrifuge tubes, followed by centrifugation (400× *g*, 10 min, 4 °C). The supernatant containing crude protein isolates was removed and transferred to fresh micro-centrifuge tubes, and protein concentration was quantified. The bicinchoninic acid assay (BCA) was used to quantify proteins, and samples were standardised to a concentration of 1.5 mg/mL. Protein samples were prepared for further usage by boiling (5 min, 100 °C) in Laemmli Buffer (distilled water, glycerol, 10% SDS, β-mercaptoethanol, 0.5 M Tris-HCl (pH 6.8), 1% bromophenol blue and glycerol).

A Bio-Rad compact supply was used to electrophorese 20 µL samples (1 h, 150 V) in sodium dodecyl sulphate (SDS) polyacrylamide gels (4% stacking, 10% resolving). Separated proteins were transferred onto nitrocellulose membranes using the Bio-Rad Trans-Blot^®^ Turbo Transfer. Blocking of membranes was carried out using 5% Bovine Serum Albumin (BSA) in Tween 20-Tris buffer saline (TBS-T: 150 mM NaCl, 3 mM KCl, 25 mM Tris, 0.05% Tween 20, dH2O, pH 7.5) for 1 h at room temperature (RT).

Membranes were then immuno-probed with the requisite primary antibody (Cell signaling Technology, Danvers, MA, USA; Phospho-IRS1 (Ser1101) Antibody (#2385T) 1:1000 dilution in 5% BSA) for 1 h at RT and overnight at 4 °C. Thereafter, membranes were washed 5 times for 10 min using 5 mL TBS-T. Membranes were then incubated in horseradish peroxidase (HRP)-conjugated secondary antibodies (Cell signaling Technology; anti-rabbit (#7074S) 1:5000 in 5% BSA) for 1 h at RT. Following incubation, membranes were washed (5 × 10 min in TBS-T) and rinsed with distilled water. Proteins were detected following the addition of Clarity Western ECL Substrate detection reagent (400 µL) (Bio-Rad, Hercules, CA, USA), and images were captured using the Bio-Rad ChemiDoc™ XRS+ Imaging System.

Membranes were quenched using 5% hydrogen peroxide for 30 min at 37 °C, blocked using 5% BSA, and incubated in HRP-conjugated antibody for β-actin (A3854, Sigma-Aldrich, St. Louis, MO, USA). β-actin is a housekeeping protein expressed evenly across cells. Image Lab™ Software version 6.0 (Bio-Rad, Hercules, CA, USA) was used for analysis of results. Relative band density of protein was calculated by normalizing results against β-actin.

### 4.4. Quantitative PCR

#### 4.4.1. RNA Isolation and Quantification

Following treatment, cells were washed using 0.1 M PBS and incubated with 500 µL Trizol and 500 µL PBS (5 min, RT). Mechanical lysis of cells was performed using a cell scraper and contents was transferred to 1.5 mL micro-centrifuge tubes and stored (24 h, −80 °C). Thereafter, samples were thawed and 100 µL chloroform was added to each tube followed by centrifugation (12,000× *g*, 10 min, 4 °C). Supernatants were removed and transferred to 1.5 mL micro-centrifuge tubes containing 250 µL. Tubes were incubated overnight at −80 °C. Following incubation, samples were thawed and centrifuged (12,000× *g*, 20 min, 4 °C). Supernatants were aspirated and discarded, and the remaining pellet was washed in 500 µL of 75 % cold ethanol followed by centrifuge (7400× *g*, 15 min, 4 °C). RNA pellets were air-dried (30 min, 24 °C) and re-suspended in 15 µL nuclease-free water. RNA quantification was carried out using Nanodrop2000 spectrophotometer (Thermo-Fisher Scientific, Waltham, MA, USA). RNA quality was determined using the A260/A280 ratio. All RNA samples were standardised to 1000 ng/µL.

#### 4.4.2. Quantification of mRNA Expression

The cDNA was synthesised from the standardised RNA samples using the iScript™ cDNA Synthesis kit as per manufacturer’s instructions (Bio-Rad, 107-8890, Hercules, CA, USA).

Transcript levels of relevant genes (Table 1) were assessed using the SsoAdvanced™ Universal SYBR^®^ Green Supermix (Bio-Rad, 1725270) and the CFX96 Touch™ Real-Time PCR Detection System (Bio-Rad, Hercules, CA, USA). The thermo-cycler conditions for each gene were as follows: initial denaturation (8 min, 95 °C), followed by 40 cycles of denaturation (15 s, 95 °C), annealing (40 s, Table 1), and extension (30 s, 72 °C). Data were normalized against the housekeeping gene, GAPDH, which is evenly expressed across cells. Results were calculated using the Livak and Schmittgen (2001) method and was represented as fold change relative to the control cells (2^−ΔΔCT^) [51].

#### 4.4.3. Quantification of miR-128a Expression

As per the manufacturer’s instructions, cDNA was synthesised using standardised RNA using the miScript II RT kit (Qiagen, 218161, Hilden, Germany). The expression of miR-128a was assessed using the miScript SYBR Green PCR Kit (Qiagen, 218073, Hilden, Germany) and CFX96 Touch™ Real-Time PCR Detection System (Bio-Rad, Hercules, CA, USA). The thermo-cycler conditions were as follows: initial denaturation (15 min, 95 °C), followed by 40 cycles of denaturation (15 s, 94 °C), annealing (30 s; 55 °C) and extension (30 s; 70 °C). Data were normalized against the housekeeping gene, *GAPDH,* which is evenly expressed across cells. Results were calculated using the Livak and Schmittgen (2001) method and was represented as fold change relative to the control cells (2^−ΔΔCT^) [51]

### 4.5. Statistical Analysis

All assays were performed in triplicate with three replicates per sample (*n* = 3). GraphPad Prism version 5.0 (GraphPad Prism Software Inc., San Diego, CA, USA) was used to perform all statistical analyses. Data were analysed using an unpaired *t*-test with data having *p* < 0.05 considered to be significant.

## 5. Future Recommendations and Limitations

The present study was carried out using HepG2 cells. HepG2 cells have commonly been used for toxicity and drug-metabolising studies due to the similarities in the physiological profile of primary hepatocytes. However, both cell lines are not identical, and it is recommended that primary hepatocytes be used in future studies to fully understand the effects of ARVs in the human liver. Additionally, other cell lines possibly derived from muscle or heart tissue can be used to support findings of metabolic syndrome in the human body. Future studies should assess similar markers in an in vivo HIV^+^ model at different exposure periods to fully understand mechanisms. Aside from this, future work should include protein analysis to determine insulin resistance initiation in the HIV^+^ in vivo model rather than the transcriptional promotion shown in the current study. We solely assessed the effect on inflammasome and insulin resistance markers; however, future studies should examine the effects of combinational ARV treatment on inflammasome activators (mitochondrial stress) to provide a better understanding of inflammasome activation. The present study assesses the expression of miR-128a as a regulator of the IRS1/AKT pathway; however, future studies need to include more markers and experiments that can contribute to understanding epigenetic modifications associated with ARV usage.

## Figures and Tables

**Table 1:** Primer sequences with respective annealing temperatures for genes assessed.

**Gene**		**Sequence (5′-3′)**	**Annealing** **Temperature (°C)**
*NLRP3*	Forward Reverse	CAGGTGTTGGAATTAGACAAC TTCAGACAACCCCAGGTTCT	60
*IL-1β*	Forward Reverse	ACGAATCTCCGACCACCACTAC TCCATGGCCACAACAACTGACG	60
*AKT*	Forward Reverse	TGGACTACCTGCACTCGGAGAA GTGCCGCAAAAGGTCTTCATGG	59
*PI3K*	Forward Reverse	GAAGCACCTGAATAGGCAAGTCG GAGCATCCATGAAATCTGGTCGC	59
*IRS1*	Forward Reverse	AGTCTGTCGTCCAGTAGCACCA ACTGGAGCCATACTCATCCGAG	59
*JNK*	Forward Reverse	GACGCCTTATGTAGTGACTCGC TCCTGGAAAGAGGATTTTGTGGC	59
*GAPDH*	Forward Reverse	TCCACCACCCTGTTGCTGTA ACCACAGTCCATGCCATCAC	---

## Data Availability

Not applicable.

## Supplementary Materials

The supporting information can be downloaded at: https://www.mdpi.com/article/10.3390/ijms24076267/s1.
