# Supplementary material for: Antiretrovirals Promote Insulin Resistance in HepG2 Liver Cells through miRNA Regulation and Transcriptional Activation of the NLRP3 Inflammasome
Source: Int J Mol Sci. 2023 Mar 27;24(7):6267. doi: 10.3390/ijms24076267 (PMC10094183; doi:10.3390/ijms24076267)
Supplement: Supplementary file 1 [file ijms-24-06267-s001.zip › ijms-2248651-supplementary.pdf]

Supplementary Data

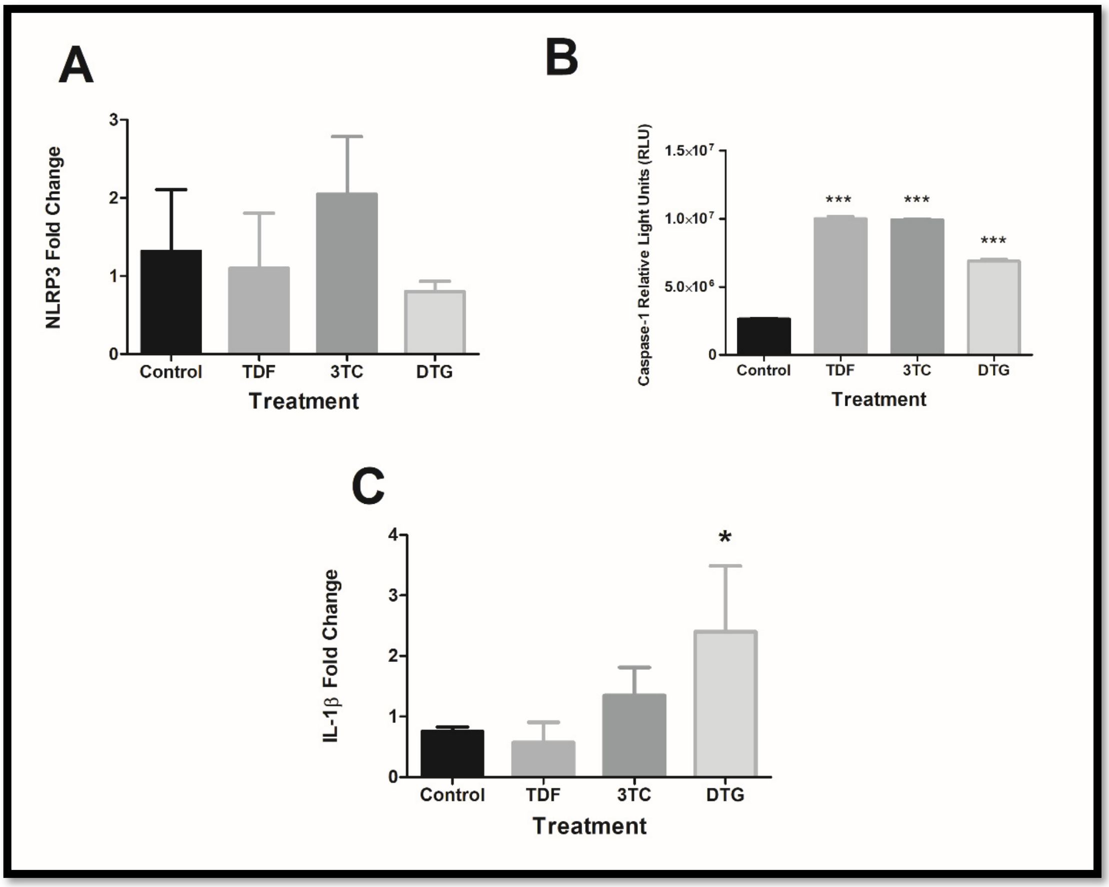

Supplementary Figure S1. Individual exposure for tested ARVs. (**A**) *NLRP3* expression; (**B**) Caspase-1 activity; (**C**) *IL-1β* expression. (p: *** $p < 0.0001$; * $p < 0.05$).

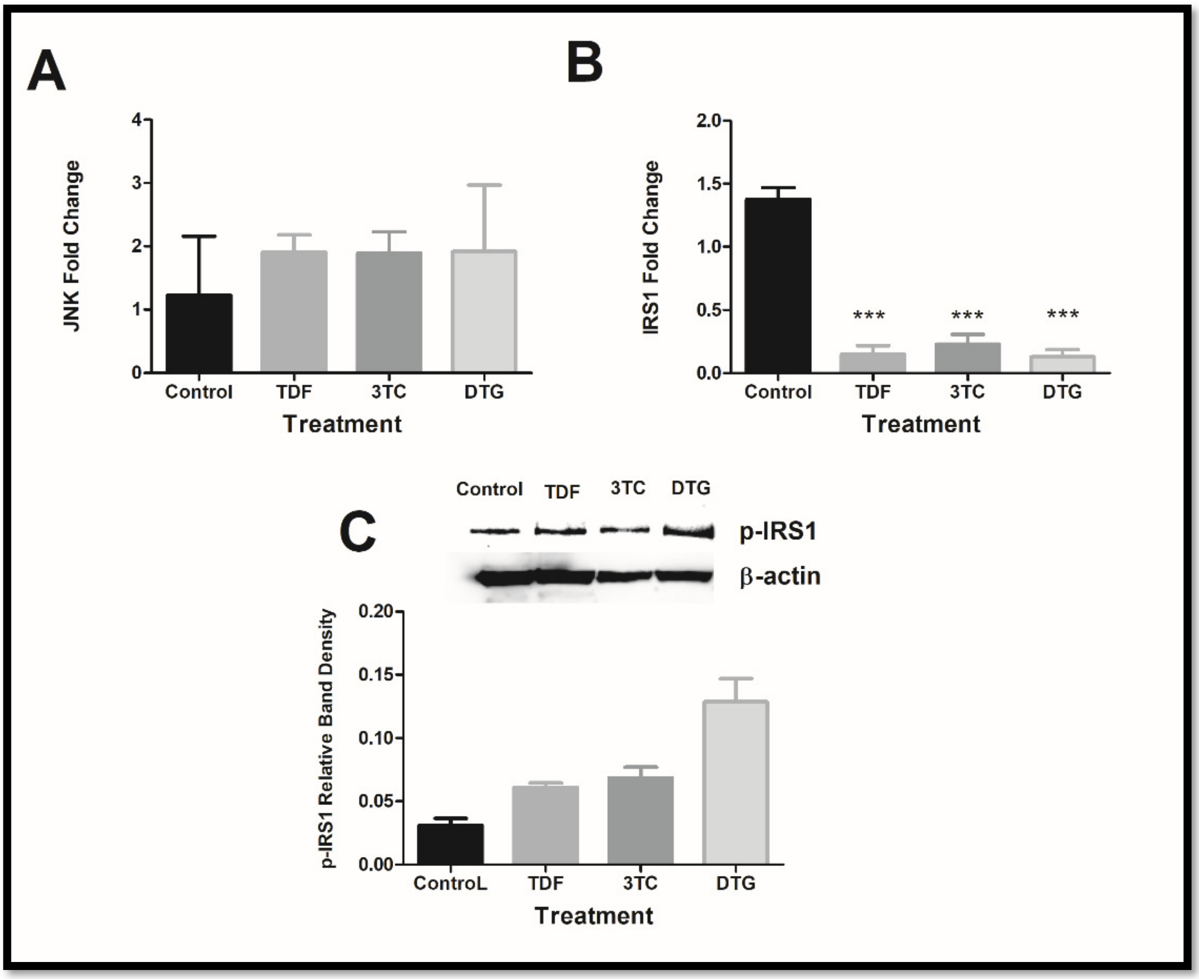

Supplementary Figure S2. Individual exposure for tested ARVs. (**A**) *JNK* expression; (**B**) *IRS1* expression; (**C**) *p*-IRS1 protein expression. (*p*: *** $p < 0.0001$).

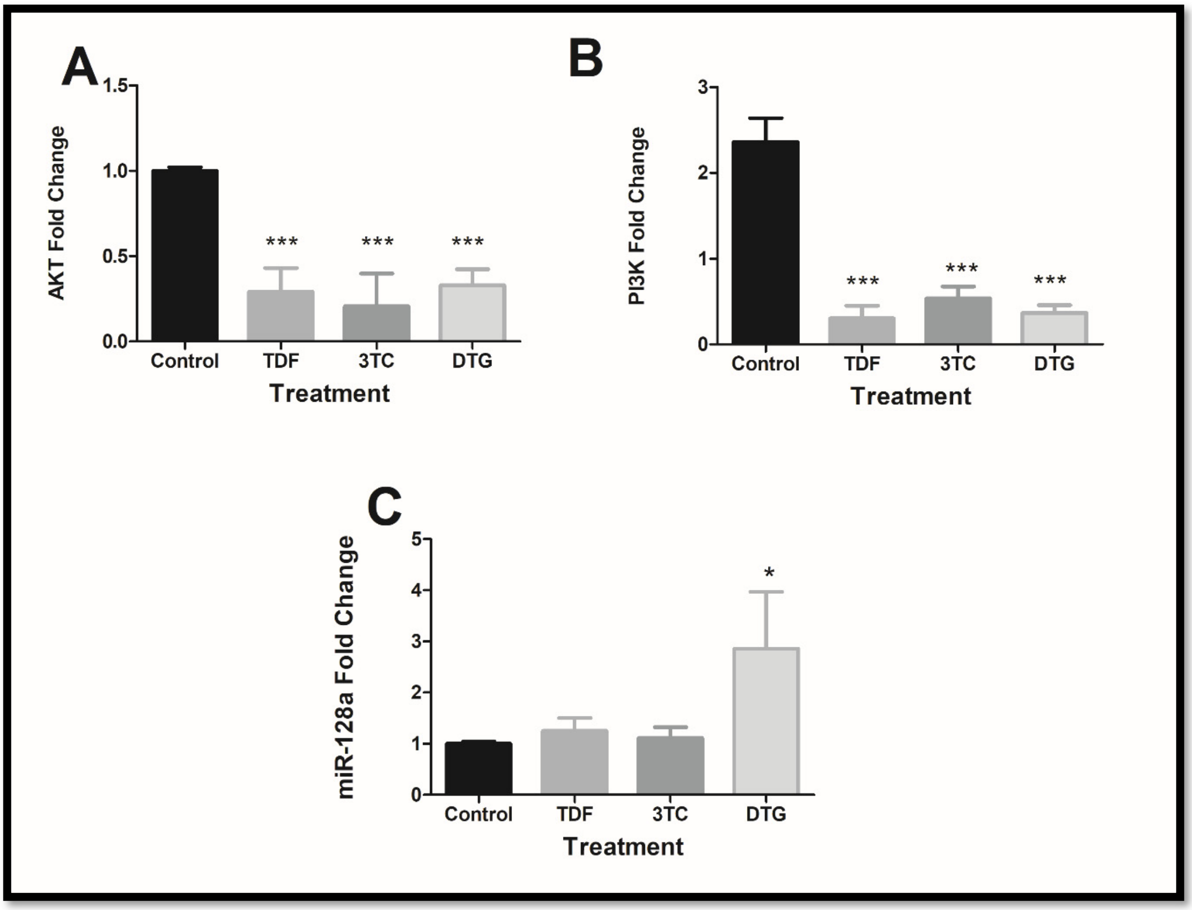

Supplementary Figure S3. Individual exposure for tested ARVs. (**A**) *AKT* expression; (**B**) *PI3K* expression; (**C**) miR-128a miRNA expression. (p: *** $p < 0.0001$; * $p < 0.05$).
